# Estimating global mortality from potentially foodborne diseases: an analysis using vital registration data

**DOI:** 10.1186/1478-7954-10-5

**Published:** 2012-03-16

**Authors:** Laura A Hanson, Elizabeth A Zahn, Sommer R Wild, Dörte Döpfer, James Scott, Claudia Stein

**Affiliations:** 1 Saint Olaf College, Northfield, MN, USA; 2 Department of Medical Sciences, School of Veterinary Medicine, University of Wisconsin, Madison, WI, USA; 3 Department of Mathematics and Statistics, Colby College, Waterville, ME, USA; 4 World Health Organization Regional Office for Europe, Copenhagen, Denmark

## Abstract

**Background:**

Foodborne diseases (FBD) comprise a large part of the global mortality burden, yet the true extent of their impact remains unknown. The present study utilizes multiple regression with the first attempt to use nonhealth variables to predict potentially FBD mortality at the country level.

**Methods:**

Vital registration (VR) data were used to build a multiple regression model incorporating nonhealth variables in addition to traditionally used health indicators. This model was subsequently used to predict FBD mortality rates for all countries of the World Health Organization classifications AmrA, AmrB, EurA, and EurB.

**Results:**

Statistical modeling strongly supported the inclusion of nonhealth variables in a multiple regression model as predictors of potentially FBD mortality. Six variables were included in the final model: *percent irrigated land, average calorie supply from animal products, meat production in metric tons, adult literacy rate, adult HIV/AIDS prevalence*, and *percent of deaths under age 5 caused by diarrheal disease*. Interestingly, nonhealth variables were not only more robust predictors of mortality than health variables but also remained significant when adding additional health variables into the analysis. Mortality rate predictions from our model ranged from 0.26 deaths per 100,000 (Netherlands) to 15.65 deaths per 100,000 (Honduras). Reported mortality rates of potentially FBD from VR data lie within the 95% prediction interval for the majority of countries (37/39) where comparison was possible.

**Conclusions:**

Nonhealth variables appear to be strong predictors of potentially FBD mortality at the country level and may be a powerful tool in the effort to estimate the global mortality burden of FBD.

**Disclaimer:**

The views expressed in this document are solely those of the authors and do not represent the views of the World Health Organization.

## Background

Foodborne diseases (FBD) encompass a wide spectrum of illnesses that manifest after the ingestion of contaminated foods and food products. They can be caused by a variety of microbial pathogens, chemicals, and parasites that contaminate food at different points in the food production and preparation process. Notwithstanding the morbidity and disability resulting from foodborne diseases, the ingestion of contaminated food can lead to death. Diarrheal diseases alone, a considerable proportion of which are foodborne, kill 1.5 million children every year worldwide [1]. Although most of these diarrheal deaths occur in poor countries, foodborne diseases are neither limited to developing countries nor to children. It is estimated that in the United States, foodborne diseases result in 37.2 million illnesses, 228,744 hospitalizations, and 2,612 deaths each year [2].

The full extent of the burden and cost of unsafe food is currently still unknown, but its impact on global health, trade, and development is considered to be immense. Recognizing the current data gap, the World Health Organization (WHO) launched the Initiative to Estimate the Global Burden of Foodborne Diseases. This Initiative seeks to use summary health metrics that combine morbidity, mortality, and disability in the form of the disability-adjusted life year (DALY). As part of this work, cause of death data from vital registration (VR) systems were analyzed to explore mortality from causes that are largely but not exclusively foodborne. The results of these analyses are presented in this paper.

## Methods

### Data sources

Mortality data for the years 2000 to 2005 were obtained from VR data collated by WHO annually from each of its member states. Causes of death (CoDs) are recorded using the International Classification of Diseases (ICD) coding system. CoDs included in this study were diseases of potentially but not exclusively foodborne origin, as ICD codes do not enable this level of detail. The diseases included were bacterial and viral gastrointestinal diseases, several parasitic diseases, and hepatitis A and E. FBD resulting from chemicals and biotoxins could not be included for lack of specific ICD codes (Table 1).

**Table 1:** Causes of death of potentially foodborne origin

Disease grouping	ICD-10 Code	Cause of death
	A01	Typhoid/Paratyphoid fever
	
	A02	Salmonella
	
	A03	Shigellosis
	
	A04	Bacterial intestinal infections
	
	A05	Bacterial foodborne intoxications
	
Diarrheal	A06	Amoebiasis
	
	A07	Protozoal intestinal diseases
	
	A08	Viral intestinal infections
	
	A09	Diarrhea and gastroenteritis of presumed infectious origin
	
	A23	Brucellosis
	
	A32	Listeriosis
	
	B15	Acute Hepatitis A

Waterborne	A00	Cholera
	
	A27	Leptospirosis

	B66	Fluke infections
	
	B67	Echinococcosis
	
Helminths	B68	Taeniasis
	
	B69	Cysticercosis
	
	B75	Trichinellosis

This study included all countries that reported at least 30 potentially FBD deaths per year and for which the WHO estimation of population coverage of VR data was at least 70%. These criteria enabled an analysis of 48 of the 96 countries included in the VR database (Table 2). For the data of these 48 countries, ICD codes were standardized to the most recent version, ICD-10, and any ill-defined or clearly miscoded CoDs were redistributed according to established practice [3].

Publicly available and validated databases, largely those from United Nations agencies, were accessed online and used to collect a wide range of explanatory variables. In addition to traditionally used health indicators, variables related to agriculture, environment, food consumption, environmental pollution, demographics, and trade were also collected and explored. Nonhealth indicators were selected based on their assumed relationship to food production, population behavior, food safety, and/or possible transmission routes of FBD. (A full list of variables collected is available upon request.)

### Calculation of mortality rates

For each of the 48 countries, mortality figures for the 19 ICD-10 codes representing potentially FBD were combined and averaged across all available years (2000-2005). Mortality rates per 100,000 were calculated using 2005 population estimates from the 2006 revision of The World Population Prospects, a publication of the UN Department of Economic and Social Affairs. Population coverage of vital events registration differed among countries; for countries with less than 100% population coverage of data, mortality rates were proportionally increased such that final mortality rates approximated 100% population coverage.

### Statistical analysis

Univariate linear regressions were performed to assess the ability of each explanatory variable to independently predict potentially FBD mortality. Each explanatory variable was regressed against the log mortality rate (log transformations were applied to satisfy linear assumptions in the regression; no outliers were noted). Variables were retained for further analyses if a relationship to mortality was indicated (α = 0.1) or if expert knowledge merited the variable's retention.

### Model construction

With a sample size of 48 countries and a large number of potential explanatory variables, it was necessary to reduce overall dimensionality during model building. In cases such as this, automated stepwise regression is sometimes used as a tool. However, an automated approach was not employed in this study due to its well-documented flaws [4] and the lack of available, complete explanatory data from all countries included in this analysis. Rather, a pragmatic approach was applied; model building proceeded via a modified nonautomated backward stepwise process. Beginning with a full model that contained all potential variables, variables were subsequently removed and replaced individually based on a consideration of standard regression outputs (t-tests, adjusted R^2^, and AIC metrics), colinearity, interaction, logic, and expert knowledge.

With a limited number of exceptions, the set of variables initially considered for inclusion in our multiple regression model were those satisfying two criteria: (1) the variable was identified as a potentially significant predictor of potentially FBD mortality based on univariate regressions (p-value < 0.1 α-level) and/or subject matter knowledge and (2) at least 40 out of the 48 countries were represented in the variable's dataset. The application of these criteria to the initial list of 91 variables narrowed the focus to 40 variables, 18 of which were traditionally used health indicators and 22 of which were designated nonhealth variables. (Additional file 1: Table A1.)

A multiple regression model was built in three steps to further aid in dimension reduction. Step 1: Multiple regression modeling was limited to the 18 traditionally used health indicators as predictors of potentially FBD mortality. Nine of these variables were identified as most likely predictive, based on t-tests using a cutoff value of α = 0.1, and retained for further modeling. Step 2: Similarly, multiple regression modeling was next limited to the 22 nonhealth variables. Eight of these variables were identified as predictive and retained for further modeling. Step 3: The 17 variables identified from the first two steps (nine traditionally used variables and eight nonhealth variables) were compiled for consideration in the final model. Modeling again proceeded according to the nonautomated approach as described above. After reduction, six variables were retained in the final model (see Additional file 1: Table A2). Residuals were generated and examined for any evidence of outliers or heteroscedasticity.

All 15 other traditionally used health indicators not remaining in the final model were again individually added to the model as a seventh variable in order to assess model robustness. Each time, any changes to the model's statistical metrics were assessed. The validity and predictive capability of the model was assessed by using it to predict FBD mortality figures for all A and B level countries in the Americas and Europe regions (AmrA, AmrB, EurA, EurB) (see Table 2), including those for which VR data were not available. Where VR mortality rates were available, these provided a comparison to the mortality rate predicted by the model. For the purpose of an additional predictive validity check, the in-sample and out-of-sample predictive validity of the final model was also investigated. A more detailed description is provided in Additional file 2. The best predictive model is expected to have robust predictions of mortality rates where the observed rates are included in the 95% prediction intervals in addition to the smallest root mean square error value.

**Table 2:** Countries included in the analysis by WHO regions and mortality level classifications

		African region	
**E**	South Africa		

		**Americas region**	

**A**	Canada	Cuba	United States

**B**	Argentina	El Salvador	Trinidad/Tobago
	
	Brazil	Guyana	Uruguay
	
	Chile	Mexico	Venezuela
	
	Columbia	Panama	
	
	Costa Rica	Paraguay	

**D**	Ecuador	Guatemala	
	
		**Eastern Mediterranean region**	

**D**	Egypt		
	
		**Europe region**	

**A**	Austria	Israel	Spain
	
	Denmark	Italy	Sweden
	
	Finland	Netherlands	Switzerland
	
	France	Norway	United Kingdom
	
	Germany	Portugal	

**B**	Bulgaria	Poland	Macedonia, FYR
	
	Georgia	Romania	Uzbekistan
	
	Kyrgyzstan	Serbia and Montenegro	

**C**	Hungary	Moldova	
	
		**Southeast Asia region**	

**B**	Thailand		
	
		**Western Pacific region**	

**A**	Australia	Japan	

**B**	Korea, South		

Countries classified as "A" are those with the lowest adult and child mortality rates. Those classified as "E" are those with the highest rates

All statistical analyses and model building were performed using STATA 9 or STATA 10.

## Results

Univariate linear regression and expert knowledge identified 17 variables as potential predictors of potentially FBD mortality. (See Additional file 1: Table A1.) Six of these variables remained in our final multiple regression model as significant predictors of potentially FBD (Table 3). Of these six variables, three are nonhealth variables (*percent irrigated land, average calorie supply from animal products*, and *meat production in metric tons*), while three are traditionally used indicators (*adult literacy rate, adult HIV/AIDS prevalence*, and *percent of deaths under age 5 caused by diarrheal diseases*). Collectively, two of the six variables are health-related, one is demographic, and three are specific to food production or consumption. This model is based on data from 47 of the 48 countries, due to literacy data being unavailable for Serbia and Montenegro. A residual-versus-fitted plot for the model showed no evidence of outliers or heteroscedasticity. Covariates included in the final model show no evidence of colinearity. *Average calorie supply from animal products *and *meat production in metric tons *were tested for interaction; an interaction term was added and found to be nonsignificant.

**Table 3:** The final predictive model incorporating both health and nonhealth indicators of potentially FBD mortality

Explanatory variable	Association	Interpretation	p-value
Irrigated land (percent)	Negative	A 1% increase in irrigated land predicts a **8.9% decrease **in FBD mortality	0.008

Calorie supply from animal products, average (kcal/person)	Negative	An increase of 10 kcals per person predicts a **2% decrease **in FBD mortality	0.018

Meat production (metric tons per 1,000 people)	Positive	An increase of 10 metric tons per 1,000 people predicts a **6% increase **in FBD mortality	0.050

Literacy rate, adults (percent)	Negative	A 1% increase in literacy predicts a **4% decrease **in FBD mortality	0.027

HIV/AIDS infection prevalence, adults (percent)	Positive	A 1% increase in HIV/AIDS predicts a **10% increase **in FBD mortality	0.067

Deaths caused by diarrheal diseases, age < 5 (percent)	Positive	A 1% increase in diarrheal deaths under age 5 predicts a **6% increase **in FBD mortality	0.078

The direction and interpretation of the association between each variable and potentially FBD mortality are indicated

From the original list of traditionally used health indicators, the fifteen not included in the final model were each added, in turn, as a seventh variable to the model. None of these fifteen variables merited inclusion based on statistical metrics. The effect of the addition of these variables on the original six variables in the model was varied. Consistently, the significance of the three food-related variables remained high, regardless of the addition of an additional heath variable. The one exception was the inclusion of *Urban population *to the model, which rendered only *Meat production *nonsignificant. Statistical metrics were considered for a model that included *Urban population *and excluded *Meat production*. However, doing so did not appear to improve the fit of model. The two health variables showed greater variability. The significance of *adult HIV/AIDS prevalence *and *percent of deaths under age 5 caused by diarrheal diseases *changed depending on which additional health covariate was added to the model. In no case did the alternative model provide statistical metrics that would suggest the rejection of the original model. This finding is supported by the predictive validity check (Additional file 2: Table A3) from which the final model proposed in Table 3 prevailed as the best predictive model in terms of both in-sample and out-of-sample predictive validity.

A country-level development indicator variable was also generated and considered for inclusion in the final model. Countries were divided based on WHO mortality-level classifications (see Table 2). Countries classified as A or B (lowest mortality; 42/48) were grouped, as were countries classified as C, D, or E (highest mortality; 6/48). This variable was not significant (p-value = 0.987, α-level) and was excluded from the model. Interaction terms between the indicator and each of the six covariates were also considered; there was no evidence for significance of these terms within the model.

Lastly, the model was used to predict FBD mortality rates for Japan, Australia, and all AmrA, AmrB, EurA, and EurB countries (Table 4). Among these countries, mortality rate predictions from our model ranged from 0.26 deaths per 100,000 (Netherlands) to 15.65 deaths per 100,000 (Honduras) in the Americas and Europe (A and B) regions. Thirty-nine of these countries were among those whose data were used for model building; predicted mortality rates for these countries were compared with VR reported mortality rates. For the majority of these countries (37/39, 95%), the predicted mortality was consistent with the reported rate (defined as the reported rate being contained within the 95% prediction interval). The two exceptions were Poland and Uruguay.

**Table 4:** FBD mortality rates as predicted by the final multiple regression model

Country	Actual rate	Predicted rate	95% PI
Albania	-	1.09	0.14, 8.72

Argentina	1.33	1.65	0.26, 10.34

Armenia	-	1.68	0.25, 11.23

Australia	0.27	1.61	0.24, 10.81

Austria	0.41	0.70	0.11, 4.58

Azerbaijan	-	1.13	0.14, 9.32

Bahamas	-	1.50	0.22, 10.03

Barbados	-	1.24	0.19, 7.89

Belgium	-	1.24	0.19, 8.13

Belize	-	3.43	0.54, 21.81

Bosnia and Herzegovina	-	2.76	0.39, 19.59

Brazil	4.20	6.73	1.03, 44.08

Bulgaria	0.52	1.03	0.17, 6.42

Canada	1.19	1.48	0.23, 9.51

Chile	1.84	1.70	0.26, 10.96

Colombia	3.88	5.32	0.83, 33.91

Costa Rica	2.96	2.08	0.33, 13.17

Croatia	-	1.75	0.26, 11.94

Cuba	2.81	1.24	0.18, 8.42

Czech Republic	-	1.31	0.21, 8.33

Denmark	1.33	1.25	0.12, 12.67

Dominican Republic	-	7.50	1.16, 48.66

Ecuador	7.51	*n/a*	*n/a*

Egypt	21.03	*n/a*	*n/a*

El Salvador	7.59	10.14	1.53, 67.23

Finland	0.89	0.70	0.11, 4.61

France	1.53	0.42	0.06, 2.91

Georgia	0.83	2.12	0.32, 13.97

Germany	0.69	0.77	0.12, 4.87

Guatemala	41.48	*n/a*	*n/a*

Guyana	13.82	8.89	1.11, 71.06

Honduras	-	15.65	2.29, 106.75

Hungary	0.35	*n/a*	*n/a*

Israel	1.66	0.78	0.12, 4.95

Italy	0.15	0.45	0.07, 2.94

Jamaica	-	6.13	0.96, 38.99

Japan	1.07	0.84	0.13, 5.48

Korea, South	0.47	*n/a*	*n/a*

Kyrgyzstan	6.96	2.48	0.37, 16.58

Malta	-	0.67	0.10, 4.39

Mexico	5.07	2.39	0.39, 14.78

Netherlands	0.49	0.26	0.03, 1.97

Norway	2.13	0.65	0.10, 4.27

Panama	4.25	5.41	0.85, 34.30

Paraguay	6.22	4.95	0.77, 31.74

Poland	0.11	1.20	0.19, 7.64

Portugal	0.16	0.58	0.09, 3.91

Macedonia, FYR	1.92	2.02	0.32, 12.68

Moldova	0.97	*n/a*	*n/a*

Romania	0.83	0.49	0.07, 3.34

Serbia and Montenegro	0.66	*n/a*	*n/a*

Slovakia	-	0.94	0.15, 5.86

Slovenia	-	1.05	0.17, 6.68

South Africa	25.84	*n/a*	*n/a*

Spain	0.94	0.77	0.12, 4.86

Suriname	-	6.80	1.03, 45.01

Sweden	0.95	0.76	0.12, 4.89

Switzerland	0.53	0.61	0.09, 4.06

Tajikistan	-	4.81	0.68, 34.15

Thailand	1.55	*n/a*	*n/a*

Trinidad/Tobago	5.04	2.65	0.38, 18.40

Turkey	-	4.92	0.76, 31.92

Turkmenistan	-	3.89	0.58, 26.27

United Kingdom	1.89	0.79	0.12, 5.06

United States	0.65	1.01	0.16, 6.32

Uruguay	29.79	2.40	0.36, 15.96

Uzbekistan	1.09	1.99	0.28, 13.99

Venezuela	6.82	5.99	0.93, 38.76

Mortality rates were predicted for countries classified by WHO as AmrA, AmrB, EurA, or EurB as well as for Japan and Australia (due to their similarities in mortality with these regions). Actual rates generated from VR data are reported for the original 48 countries included in this study and have been adjusted for population coverage. All rates are reported as deaths per 100,000. The 95% prediction interval (PI) is reported as lower limit, upper limit

## Discussion

Foodborne diseases continue to be a major contributor to morbidity and mortality worldwide [1]. The wide range of illnesses and the presence of multiple routes of transmission make computing epidemiological estimates of the burden of FBD a challenging task. Regardless, reliable mortality estimates are crucial to the World Health Organization's strategy to reduce this burden. The present study, part of WHO's Initiative to Estimate the Global Burden of Foodborne Disease, sought to assess of the utility of nonhealth-related variables as predictors of potentially FBD mortality. Through the model building process, numerous insights were gained.

The most significant finding from this analysis is that so-called nonhealth variables improve the estimation of FBD mortality. Specifically, some nonhealth variables have proved to be more prominent predictors of potentially FBD mortality than some of the traditionally used health indictors. As demonstrated by this analysis, only two of the six variables included in the final model were directly related to health. The four nonhealth variables proved to be robust predictors of potentially FBD mortality, remaining significant even with the addition of a number of other traditionally used health indicators. Health indicators do not therefore appear to be confounding variables in this model. As such, we propose that nonhealth variables are likely providing unique and previously unmodeled information in FBD estimations.

While health indicators will always remain strong predictors of overall health outcomes, food-related variables may provide more specific insights into estimating outcomes of potentially FBD. Model building for this analysis included testing nonhealth variables from a variety of categories that were both food and nonfood related. However, all of the nonhealth variables that remained in the final model could be specifically linked to food production or consumption.

Furthermore, the nonhealth variables that appear in the final model make intuitive sense. For example, increased *meat production *predicts increased FBD mortality, which could be related to unsafe handling practices. A higher per capita *average calorie supply from animal products *predicts reduced mortality and could be an indication of improved nutrition. Likewise, increased *percent irrigated land *predicts reduced mortality, perhaps due a higher availability of safe food supplies. Case studies regarding these and other predictive variables may aid in the confirmation and discovery of contributors to the global mortality burden of FBD.

This analysis also provides support for the use of nonhealth variables in predicting potentially FBD mortality in countries lacking VR data. However, nonhealth models should not be used indiscriminately. In this analysis, 42 of the 48 countries were classified in the two lowest mortality levels (A or B). Additionally, the geographic distribution of these countries was skewed heavily in favor of the Americas and Europe regions. We suspect this grouping of countries to be somewhat self-selected. That is, countries with better reporting of FBD tend to be more developed and have demonstrated a lower incidence of FBD. The predictive capabilities of our model are therefore strongest for countries at a similar development level and with comparable FBD incidence. As such, we focused on predicting potentially FBD mortality in AmrA, AmrB, EurA, and EurB region countries.

Recent estimates from county-level studies of FBD mortality were consistent with predictions from our model. A 2011 study from the United States estimated the number of deaths from foodborne infections at approximately 2,612 each year (90% credible interval 1,723-3,819) [2]. This compares favorably with the predicted number of deaths from our model at 3,058 deaths per year with an upper limit of 19,135 deaths per year (rate of 1.01 per 100,000, upper limit 6.32 per 100,000). In the Netherlands, a national study of the burden of FBD concluded that approximately 80 persons die from foodborne infections every year [5]; our model again predicts well, estimating 43 deaths per year with an upper limit of 326 persons per year. Given the known problems of miscoding and misclassification inherent to CoD registration data, particularly in diseases that are not frequently observed, we assume that VR has underestimated the true incidence of potentially FBD mortality.

The predictive accuracy of nonhealth variables was supported by the validity of the FBD estimates generated by our model. The results of the predictive validity check in Additional file 2: Table A3 were consistent with our final model choice. Specifically, we found that our final model had good out-of-sample predictive validity (i.e., our model yielded smaller prediction errors than did other candidate models when predicting data points outside the original data set). We suspect that factors such as geographic location, cultural practices, and economic status are likely to have a strong effect on which particular nonhealth indicators are predictive of FBD mortality. Therefore it is hoped that further model building efforts will focus on specific regions or development levels. However, the lack of available data in these areas provides an obstacle to this aim, further underscoring the need for improved data collection and reporting in countries with high mortality rates.

## Conclusion

Foodborne diseases are a global problem, causing considerable morbidity and mortality annually. In this study, we have shown the potential value of using nonhealth variables alongside traditionally used health variables to predict potentially foodborne disease mortality at the country level. Our analyses were limited by the paucity of vital registration data in certain regions, but nevertheless demonstrate the predictive strength of nonhealth variables. Moreover, in addition to enhancing the specificity of cause of death modeling, nonhealth predictors could provide practical alternative measures of foodborne disease mortality in countries where traditionally used indicators may not be available or measured. Such analyses could provide valuable insight into the cause and source of disease at the country level, informing subsequent policies and interventions. As such, the expansion of the traditional health model to include nonhealth variables has the potential to be a powerful new tool in disease burden studies, including the current effort by WHO to estimate the global burden of foodborne disease.

## Competing interests

The authors declare that they have no competing interests.

## Authors' contributions

LAH and EAZ participated in study design, compiled the data, carried out statistical modeling and analysis, and helped draft the manuscript. SRW performed preliminary statistical modeling and assisted in manuscript revision. DD and JS assisted in study design, statistical analysis, and manuscript revision. CS conceived of the study, participated in study design, and helped draft the manuscript. All authors read and approved the final manuscript.

## Supplementary Material

Additional file 1 **Table A1: Descriptions and summary statistics for the 40 explanatory variables considered during model building**. Table A2: Statistical summary of final predicative model. Click here for file

Additional file 2 **Table A3: Outcomes for the predictive validity check using four competing models compared to the predictions of the final model proposed in the manuscript**. Click here for file
