# Supplementary material for: Estimating global mortality from potentially foodborne diseases: an analysis using vital registration data
Source: Popul Health Metr. 2012 Mar 16;10:5. doi: 10.1186/1478-7954-10-5 (PMC3341201; doi:10.1186/1478-7954-10-5)
Supplement: Additional file 1 — Table A1: Descriptions and summary statistics for the 40 explanatory variables considered during model building. Table A2: Statistical summary of final predicative model. [file 1478-7954-10-5-S1.PDF]

Appendix

Table A1. Descriptions and summary statistics for all forty explanatory variables considered during model-building. P-values are provided for each variable regressed independently against FBD mortality. The total number of countries in this study was 48; many variable datasets lacked at least one country. Sources are as follows: DESA: UN Department of Economic and Social Affairs†; IAEA: International Atomic Energy Agency (http://nucleus.iaea.org/Home/index.html#); ILO: International Labour Organization†; FAO: Food and Agriculture Organization†; UNDP: UN Development Programme (http://hdrstats.undp.org/); UNSD: UN Statistics Division†; WHO: World Health Organization (http://www.who.int/whosis/en); PRB: United States Population Reference Bureau (http://www.prb.org/DataFinder.aspx).

Variable Name	Units	Source	Year	n	Mean	SD	P-value	Coefficient	SE	95% CI
Population density	people per 1000 sq km	DESA	2005	47	108.5	110.2	0.082	-0.003	0.002	-0.007, 0.0004
Approval to irradiate some food item	yes/no	IAEA	n/a	48	n/a	n/a	0.044	-0.839	0.405	-1.654, -0.024
Economically active population	percent of population	ILO	2005	44	43.6	6.97	0.005	-0.086	0.029	-0.144, -0.029
Agriculture import value, total	$USD per capita	FAO	2005	47	316.1	366.8	0.098	-0.001	0.001	-0.003, -0.0004
Calorie supply from animal products, average	Kcal per person	FAO	2002	48	744.4	321.3	<0.001	-0.003	0.001	-0.004, -0.002
Cereals production	metric tons per 1000 people	FAO	2005	48	477.4	478.4	0.020	-0.001	0.0004	-0.002, -0.0002
Drylands area	percent of total land area	FAO	*	47	25.2	31.99	0.408	-0.005	0.006	-0.018, 0.008
Drylands, population in	percent of population	FAO	*	48	22.8	31.9	0.899	-0.001	0.006	-0.014, 0.012
Economically active population in agriculture	percent of population	FAO	2000	47	43.6	6.97	0.005	-0.086	0.029	-0.144, -0.028
Fishery imports of 7 fishery commodity groups	$USD per capita	FAO	2005	47	41.0	78.2	0.092	-0.004	0.003	-0.009, 0.001
Forestry import value	$USD per capita	FAO	2005	48	95.6	108.1	0.003	-0.005	0.002	-0.009, -0.002
Irrigated Land	percent of total land area (0-1)	FAO	2002	48	0.039	0.042	0.036	-9.97	4.62	-19.3, -0.658
Livestock disease – Bovine Spongiform Encephalopathy	Indicator of presence	FAO	2004	47	n/a	n/a	0.000	0.263	0.068	0.125, 0.400
Livestock disease - Foot and Mouth Disease	Indicator of presence	FAO	2004	48	n/a	n/a	0.090	-0.187	0.108	-0.405, 0.030
Meat production	metric tons per 1000 people	FAO	2005	48	71.8	63.2	0.093	-0.005	0.003	-0.012, 0.001
Protein consumption	grams per person/day	FAO	2003	48	88.9	19.6	0.000	-0.038	0.009	-0.056, -0.020
Secure tenure, households without access to	percent of urban population	UNSD	2001	47	16.5	15.8	<0.001	0.048	0.011	0.026, 0.071
Solid fuels, population using	percent of population	UNSD	2003	43	17.9	21.4	0.007	0.026	0.009	0.008, 0.045
Consumer Price Index	-	World Bank	2003	43	118.2	20.1	0.046	0.022	0.010	0.0004, 0.043

Gross National Income (GNI)	$USD per capita, in millions	World Bank	2005	44	13347	13907	0.002	-0.00005	0.00001	-.0001, -.00002
Economically active females, ages 15+	percent	PRB	95-02	47	50.2	10.8	0.015	-0.046	0.018	-0.082, -0.009
Motor vehicles	units per 1000 people	PRB	00-05	40	345.9	230.2	<0.001	-0.004	0.001	-0.005, -0.002
**HEALTH VARIABLES**										
Literacy rate, adults	percent	UNDP	2001	47	94.9	8.14	<0.001	-0.103	0.020	-0.144, -0.061
GDP	$USD per capita	UNSD	2000	47	11518	11729	0.003	-0.00005	0.00002	-.0001, -.00002
HIV/AIDS infection prevalence, adults	percent	UNSD	1999	48	0.81	2.9	0.016	0.167	0.067	0.033, 0.301
Improved sanitation, population with access to	percent	UNSD	2004	44	89.6	13.4	<0.001	-0.058	0.013	-0.084, -0.032
Improved water sources, population with access to	percent	UNSD	2004	46	94.0	8.5	0.067	-0.043	0.023	-0.090, 0.003
Measles immunization coverage of 1 year olds	percent	UNSD	2003	48	92.1	7.05	0.444	-0.022	0.029	-0.080, 0.0358
Nutrition; dietary energy supply	Kcal per person/day	UNSD	98-00	29	2770	340.0	0.034	-0.002	0.001	-0.003, -0.0001
Nutrition; undernourished population	percent of population	UNSD	2003	47	5.83	7.34	0.003	0.077	0.025	0.027, 0.127
Urban population	percent of population	UNSD	2000	48	66.7	16.6	0.250	-0.014	0.012	-0.039, 0.010
Deaths caused by diarrheal diseases, age <5	percent of deaths	WHO	2000	48	4.59	5.79	<0.001	0.136	0.029	0.077, 0.194
DTP3 immunization coverage of 1 year olds	percent	WHO	2004	48	93.4	5.43	0.086	-0.064	0.037	-0.138, 0.009
Health expenditure, total	$USD per capita	WHO	2004	47	1450	1726	0.002	-0.0003	0.0001	-0.001, -0.0001
Health expenditure, total; as percent of GDP	percent of GDP	WHO	2004	47	7.73	2.22	0.017	-0.218	0.088	-0.396, -0.041
Health expenditure of government, total	$USD per capita	WHO	2004	47	1017	1234	0.002	-0.0005	0.0002	-.0008, -.0002
Infant mortality rate	per 1000 live births	WHO	2004	48	15.37	14.9	<0.001	0.052	0.011	0.029, 0.075
Life expectancy at birth	years	WHO	2004	48	75.1	5.9	<0.001	-0.133	0.028	-0.190, -0.076
Nurse density	per 1000 people	WHO	2004	47	5.77	4.04	0.002	-0.150	0.046	-0.243, -0.057
Physician density	per 1000 people	WHO	2005	48	2.4	1.17	<0.001	-0.612	0.150	-0.913, -0.310

[†] Accessed through United Nations Environment Programme (UNEP) Global Environment Outlook Database (http://geodata.grid.unep.ch/)
* listed as “various” in database

Table A2. Statistical summary of final predictive model.

	Coefficient	Std. Error	t	P>|t|	[95% Conf. Interval]	
Irrigated Land (percent)	-9.280224	3.341336	-2.78	0.008	-16.03332	-2.527132
Calorie supply from animal products, average (kcal/person)	-.0018535	.0007504	-2.47	0.018	-.0033702	-.0003369
Meat production (metric tons per 1000 people)	.0056004	.0027735	2.02	0.050	-5.04e-06	.0112058
Literacy rate, adults (percent)	-.0447247	.0195461	-2.29	0.027	-.0842288	-.0052206
HIV/AIDS infection prevalence, adults (percent)	.0912223	.0484899	1.88	0.067	-.0067794	.1892241
Deaths caused by diarrheal diseases, age <5 (percent)	.0581747	.032209	1.81	0.078	-.0069222	.1232716
Constant	5.816933	1.815988	3.20	0.003	2.146683	9.487182
