# Supplementary material for: Estimating global mortality from potentially foodborne diseases: an analysis using vital registration data
Source: Popul Health Metr. 2012 Mar 16;10:5. doi: 10.1186/1478-7954-10-5 (PMC3341201; doi:10.1186/1478-7954-10-5)
Supplement: Additional file 2 — Table A3: Outcomes for the predictive validity check using four competing models compared to the predictions of the final model proposed in the manuscript. [file 1478-7954-10-5-S2.DOC]

Strategy for the Predictive Validity Check

A total of five additional models were constructed to investigate the predictive validity of our proposed model. For each model, predicted mortality rates for the USA and The Netherlands were calculated along with corresponding 95% prediction intervals as well as the RMSE (Table A3). Three specific comparisons were made: (i) Comparison 1: the proposed model with six predictors was compared to the proposed model with one predictor removed. (ii) Comparison 2: data from The Netherlands was removed. Subsequently, the predicted mortality for The Netherlands using the proposed model with six predictors was compared to the predicted mortality using the proposed model with one predictor removed. (iii) Comparison 3: data from the United States was removed. Subsequently, the predicted mortality for the United States using the proposed model with six predictors was compared to the predicted mortality using the proposed model with one predictor removed.

Results supported our final model choice. Specifically, the RMSE’s from Comparison 1 reveal that the inclusion of an additional variable improves model fit, as would be expected, slightly (by approximately 3%). Out-of-sample validity was assessed using results from Comparisons 2 and 3. In both comparisons, the model that included all predictor variables produced predicted mortalities that were closer to those actually observed (e.g. in Comparison 2 the predicted mortality value for The Netherlands using the full model, 0.20, is closer to the observed value of 0.49 than is the predicted value when one variable is removed, 0.17).

Table A3: observed and predicted mortality rates, their 95% prediction intervals, and Root-MSE values of 6 source models

| Model | Observed | Predicted | 95% prediction interval | RMSE of source model |
| --- | --- | --- | --- | --- |
| Comparison 1 |  |  |  |  |
| Final proposed model | NL : 0.49  USA: 0.65 | 0.26  1.01 | 0.03 - 1.97  0.16 - 6.32 | 0.892 |
| Final proposed model without 1 predictor* | NL : 0.49  USA: 0.65 | 0.23  1.06 | 0.03 - 1.81  0.16 - 7.00 | 0.916 |
| Comparison 2 (data without The Netherlands) | | |  |  |
| Final proposed model | NL : 0.49 | 0.20 | 0.02 - 1.67 | 0.895 |
| Final proposed model without 1 predictor* | NL : 0.49 | 0.17 | 0.02 - 1.46 | 0.916 |
| Comparison 3 (data without the USA) | | |  |  |
| Final proposed model | USA: 0.65 | 1.03 | 0.16 - 6.59 | 0.901 |
| Final proposed model without 1 predictor* | USA: 0.65 | 1.09 | 0.16 - 7.31 | 0.924 |

* deaths due to childhood diarrhea under the age of 5 years
